# Different managements for prepubertal epididymitis based on a preexisting genitourinary anomaly diagnosis

**DOI:** 10.1371/journal.pone.0194761

**Published:** 2018-04-18

**Authors:** Yong Seung Lee, Sang Woon Kim, Sang Won Han

**Affiliations:** Department of Urology and Urological Science Institute, Yonsei University College of Medicine, Seoul, Republic of Korea; Universite Clermont Auvergne, FRANCE

## Abstract

There is no clear consensus regarding investigating for accompanying genitourinary anomalies (GUAs) in patients with prepubertal acute epididymitis (AE). Moreover, risk factors for the recurrence and the need for a surgical intervention have never been discussed. The purpose of this study was to evaluate the different clinical courses of prepubertal AE based on knowledge of preexisting GUAs. Between January 2005 and December 2014, AE was diagnosed in 189 pediatric patients <10 years old. Clinical characteristics and treatments were retrospectively analyzed. The median age at first AE was 64.3 months. A GUA was detected prior to the development of AE in 49 patients (known GUA group) including 34 with hypospadias. Among the other 140 patients (unknown GUA status group), six patients were diagnosed with a GUA after the first AE episode. In the known GUA group, 35 patients (71.4%) experienced recurrence and the only risk factor associated with recurrence was the presence of cystic dilated prostatic utricle (p = 0.013). In the unknown GUA status group, the risk factors for an existing GUA were being <1-year-old (p<0.001) and positive urine culture (p = 0.015). Only nine patients (6.4%) in this group experienced recurrence. Vasectomy was recommended for patients with recurrent AE with an accompanying GUA and performed in 19 patients (10.1%). Most GUAs are diagnosed prior to AE development. Clinicians should consider different treatment approaches based on whether the AE patient has been diagnosed with a GUA previously, because the clinical characteristics and the recurrence rate are significantly different.

## Introduction

Acute epididymitis (AE) is one of the most common causes of acute scrotum.[1] Pediatric AE has an annual incidence of approximately 1.2 per 1000 male children. Unlike postpubertal AE, accompanying genitourinary anomalies (GUAs) are known to be major causes of prepubertal AEs.[2, 3] Most previous studies regarding prepubertal AEs focused primarily on the presence of a GUA, but without any consensus regarding the assessment of GUAs after the first AE episode.[2–4] In addition, some clinicians believe that investigating for an accompanied GUA after the first AE episode may have little clinical benefit, because most GUAs are detected prior to the first AE episode. Moreover, to the best of our knowledge, no studies have investigated the benefit of further GUA evaluation looking for hidden anomalies in patients with known GUAs, the risk factors for AE recurrence in prepubertal patients, and the appropriate time to perform a vasectomy.

Herein, we evaluated the different clinical characteristics associated with prepubertal AE with respect to known and unknown GUA status to understand the different clinical course in treating prepubertal AEs.

## Materials and methods

We performed a retrospective cohort analysis of patients who were diagnosed with AE in our institution, were less than 10 years old, and had received more than 12-months follow up. This study was approved by the Institutional Review Board of Severance Hospital, Seoul, Republic of Korea (4-2015-0549). The Institutional Review Board waived the need for consent and all data was accessed anonymously.

### Data collection

Between January 2005 and December 2014, 189 patients met criteria. The age at the first AE episode, laterality, fever, urine culture, accompanying GUA, the timing of the GUA diagnosis with respect to the AE diagnosis, recurrence, and treatment strategy were analyzed. AE was diagnosed by physical examination and Doppler ultrasonography at the first episode using the following criteria: localized enlargement and epididymal tenderness during the physical and increased epididymal size and blood flow on Doppler ultrasonography.[4]

When there was a clear sign of testicular appendix torsion such as ‘blue dot sign’ on physical examination or swelling and ischemic sign of testicular appendix on ultrasonography, it was excluded.

### Statistical analysis

Univariate analyses were performed using the Fisher’s exact test and the Mann-Whitney *U* test in the SPSS software, version 18.0 (SPSS Inc., Chicago, IL). P-values <0.05 were considered statistically significant.

## Results

**Fig 1:**
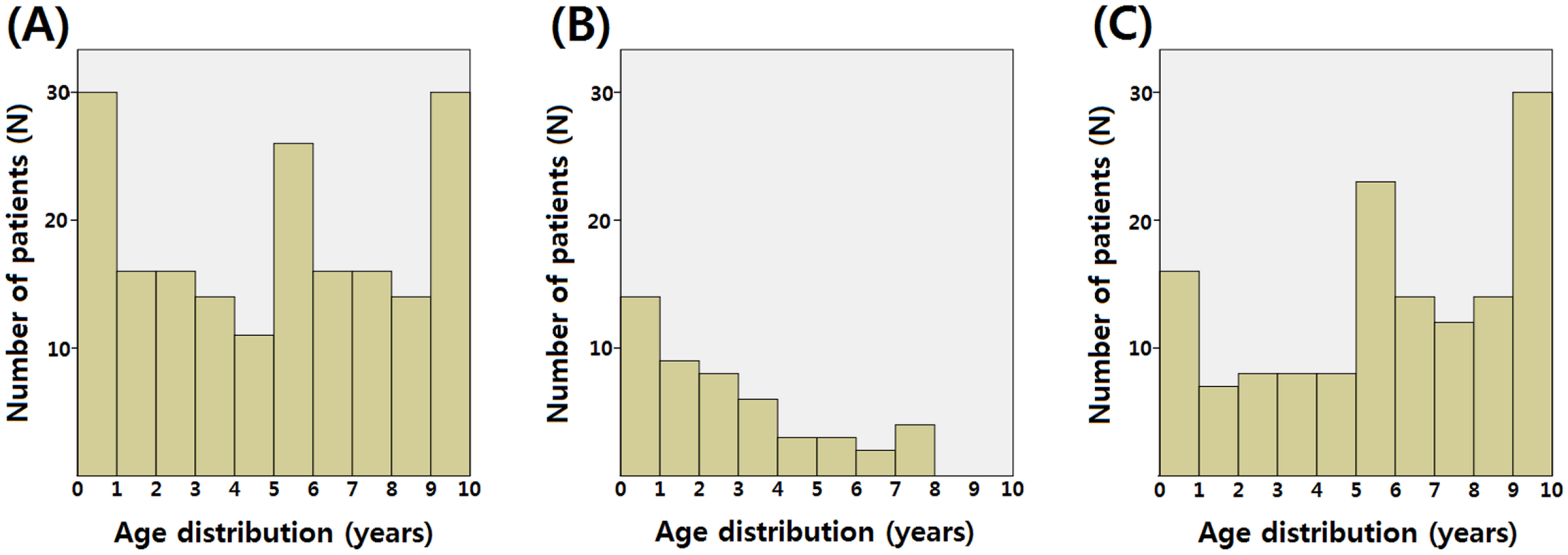
Age distribution of prepubertal epididymitis in all patients (A), in the known genitourinary anomaly group (B), and in the unknown genitourinary anomaly status group (C).

Patient characteristics are shown in Table 1. Median age at first AE episode was 64.3 months. The two most common time periods for diagnosis were from 0–1 year old and 9–10 years old (both 15.9% of patients) (Fig 1A). An accompanying GUA was observed in 55 patients (29.1%). Of those, 49 (89.1%) were detected prior to the first AE episode, whereas 6 (10.9%) were detected following the first AE episode. Patients diagnosed with GUA prior to the first AE episode were designated as the known GUA group, and all other patients were designated as the unknown GUA status group. There were statistically significant differences between the two groups in the following baseline characteristics: age at first AE episode, positive urine culture, recurrence, and vasectomy performed (Table 2).

**Table 1:** Patient characteristics (N = 189).

Variables	N (%)
Median age at first epididymitis (months)	64.3 (IQR: 24.5–92.5)
Laterality	
Unilateral (R:L)	175 (81:94)
Bilateral	14
Fever at diagnosis (> 38°C)	21 (11.1)
Positive urine culture at diagnosis*	28 (45.2*)
Accompanying anomaly	55 (29.1)
Hypospadias	34 (18.0)
Distal	14
Penoscrotal	9
Proximal	11
Presence of the cystic dilated prostatic utricle	27 (14.3)
Anorectal malformation	9 (4.8)
Upper urinary tract anomaly	9 (4.8)
Neurogenic bladder	6 (3.2)
Recurrence	44 (23.3)
Median number of episodes^†^	3 (IQR: 1–3)
Median interval between each episode (months) ^†^	3.6 (IQR: 1.6–8.0)
Vasectomy	19 (10.0)

*among 62 patients with urine culture results available;

^†^among 44 patients with recurrence

**Table 2:** Comparison of clinical parameters between AE patients with known genitourinary anomalies and those with unknown genitourinary anomaly status (N = 189).

Variables	Unknown GUA status (n = 140)	Known GUA (n = 49)	p-value
Age at initial diagnosis (months)	72.0 (IQR: 43.5–104.0)	27.2 (IQR: 10.9–46.9)	<0.001
Epididymitis before 1 year old	16	14	0.011
Laterality			0.052
Unilateral	133	42	
Bilateral	7	7	
Fever at diagnosis (> 38°C)	15	6	0.794
Positive urine culture at diagnosis*	6/32	22/30	<0.001
Accompanying anomaly	6	49	<0.001
Recurrence	9	35	<0.001
Vasectomy	2	17	<0.001

IQR, interquartile range; GUA, genitourinary anomaly;

*Shown as (number of positive cultures/total number of cultures analyzed)

### The known genitourinary abnormality group

In 49 patients with known GUA group, the age at which the patient experienced their first AE episode most frequently occurred when the patient was 0–1 year old (28.6%), followed by 1–2 years old (18.4%) (Fig 1B). The type of GUAs included 34 hypospadias, nine anorectal malformations, eight upper urinary tract abnormalities, six cystic dilated prostatic utricles (CDPU), and six neurogenic bladders. Further analysis after the first AE episode revealed additional CDPUs and an upper urinary tract abnormality in 15 and one patients, respectively.

Prostatic utricles were diagnosed using ultrasonography, voiding cystourethrography (VCUG), retrograde urethrography (RGU), computerized tomography, and magnetic resonance imaging. Among the 21 patients with CDPU, ultrasonography was performed in 13 patients, and 10 patients (76.9%) had an apparent enlarged CDPU. VCUG and RGU were performed in 10 and 11 patients, respectively, and revealed CDPUs in six (60.0%) and seven patients (63.6%), respectively. When VCUG and RGU were combined, the detection rate increased to 78.6% (11 out of 14 patients). In two patients, imaging methods, including VCUG, RGU, and ultrasonography, were negative for CDPUs; however, they were later identified during cystoscopic evaluation.

Of the 34 hypospadias patients, the AE episode occurred prior to the correction of the hypospadias in three patients (8.8%), whereas in the other 31 patients (91.2%), it occurred following the correction. Additional operations were needed in 19 hypospadias patients (55.9%) due to stenosis or fistula.

With respect to anorectal malformation, all nine patients presented with imperforated anuses and rectourethral fistulas. The AE episode occurred prior to rectourethral fistula correction in two patients (22.2%), and following correction in the other seven patients (77.8%). Among the nine upper urinary tract anomaly patients, five patients (55.6%) presented with vesicoureteral reflux, including one case of ectopic vas insertion into the ureter. Multicystic dysplastic kidney was observed in two patients (22.2%), including one case of suspected ectopic ureter insertion into the seminal vesicle. Ectopic ureter insertion was observed in two patients (22.2%), which included a single-system ectopic ureter insertion and a duplex-system with ectopic ureterocele.

**Fig 2:**
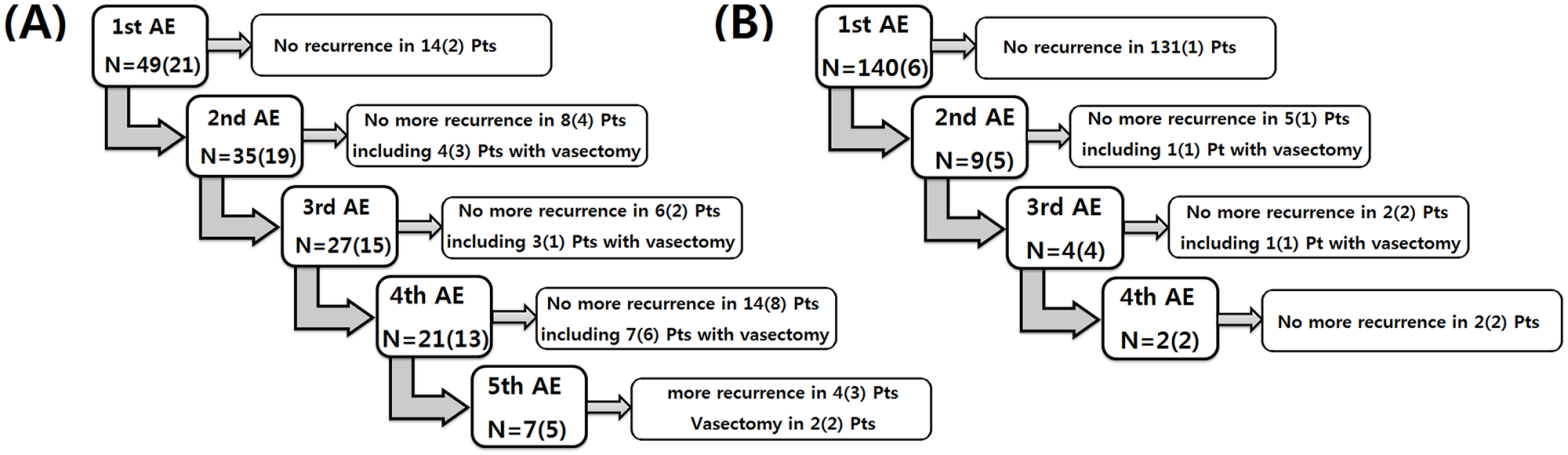
Recurrence of epididymitis in the known genitourinary anomaly group (A), and the unknown genitourinary anomaly status group (B). The number in the parenthesis is the number of patients with cystic dilated prostatic utricles; AE, acute epididymitis; Pt, patient.

Thirty-five patients (71.4%) experienced a second AE episode, and four patients underwent a vasectomy after this episode (Fig 2A). Twenty-seven of the 31 nonvasectomized patients (87.1%) experienced a third AE episode. The only observed risk factor for recurrence was the presence of a CDPU (p = 0.013) (Table 3).

**Table 3:** Comparison of clinical parameters between patients with single episode of epididymitis and recurrent epididymitis in patients with known genitourinary anomalies (N = 49).

Variables	Single episode (n = 14)	Recurrent (n = 35)	p-value
Age at initial diagnosis (months)	34.4 (IQR: 11.2–68.0)	23.9 (IQR: 9.9–40.6)	0.419
Epididymitis before 1 year old	4	10	>0.999
Laterality			0.656
Unilateral	13	29	
Bilateral	1	6	
Fever at diagnosis	1	5	0.659
Positive urine culture at diagnosis*	3/5	19/25	0.589
Accompanying anomaly			
Hypospadias	10	24	>0.999
Presence of the cystic dilated prostatic utricle	2	19	0.013
Anorectal malformation	1	8	0.415
Upper urinary tract anomaly	3	6	0.702
Neurogenic bladder	0	6	0.164

IQR, interquartile range;

*Shown as (number of positive cultures/total number of cultures analyzed)

### The unknown genitourinary abnormality status group

The remaining 140 patients that experienced AE episodes had not been evaluated for a GUA. In these patients, the most common age at first AE episode was 9–10 years old (21.4%) (Fig 1C). After the first AE episode, an accompanying GUA was diagnosed in six patients (4.3%), and all of those cases were CDPUs. All of the utricles were detected using ultrasonography. In addition, three of the CDPU patients were also evaluated with VCUG; however, VCUG was unable to detect the abnormality. The risk factors for having a GUA were having an AE episode when 0–1 year old (p<0.001) and having a positive urine culture (p = 0.015) (Table 4). Nine patients (6.4%) experienced AE episode recurrence (Fig 2B). Four patients experienced a third AE episode, and all four had enlarged CDPUs.

**Table 4:** Comparison of clinical parameters between AE patients with and without genitourinary anomalies in the unknown genitourinary anomaly status group (N = 140).

Variables	Without anomaly (n = 134)	With anomaly (n = 6)	p-value
Age at initial diagnosis (months)	74.1 (IQR: 49.1–104.6)	4.5 (IQR: 2.8–7.3)	<0.001
Epididymitis before 1 year old	10	6	<0.001
Laterality			>0.999
Unilateral	127	6	
Bilateral	7	0	
Fever at diagnosis (> 38°C)	14	1	0.500
Positive urine culture at diagnosis*	3/28	1/4	0.015
Recurrence	4	5	<0.001

IQR: interquartile range,

*Shown as (number of positive cultures/total number of cultures analyzed)

### Treatment

**Fig 3:**
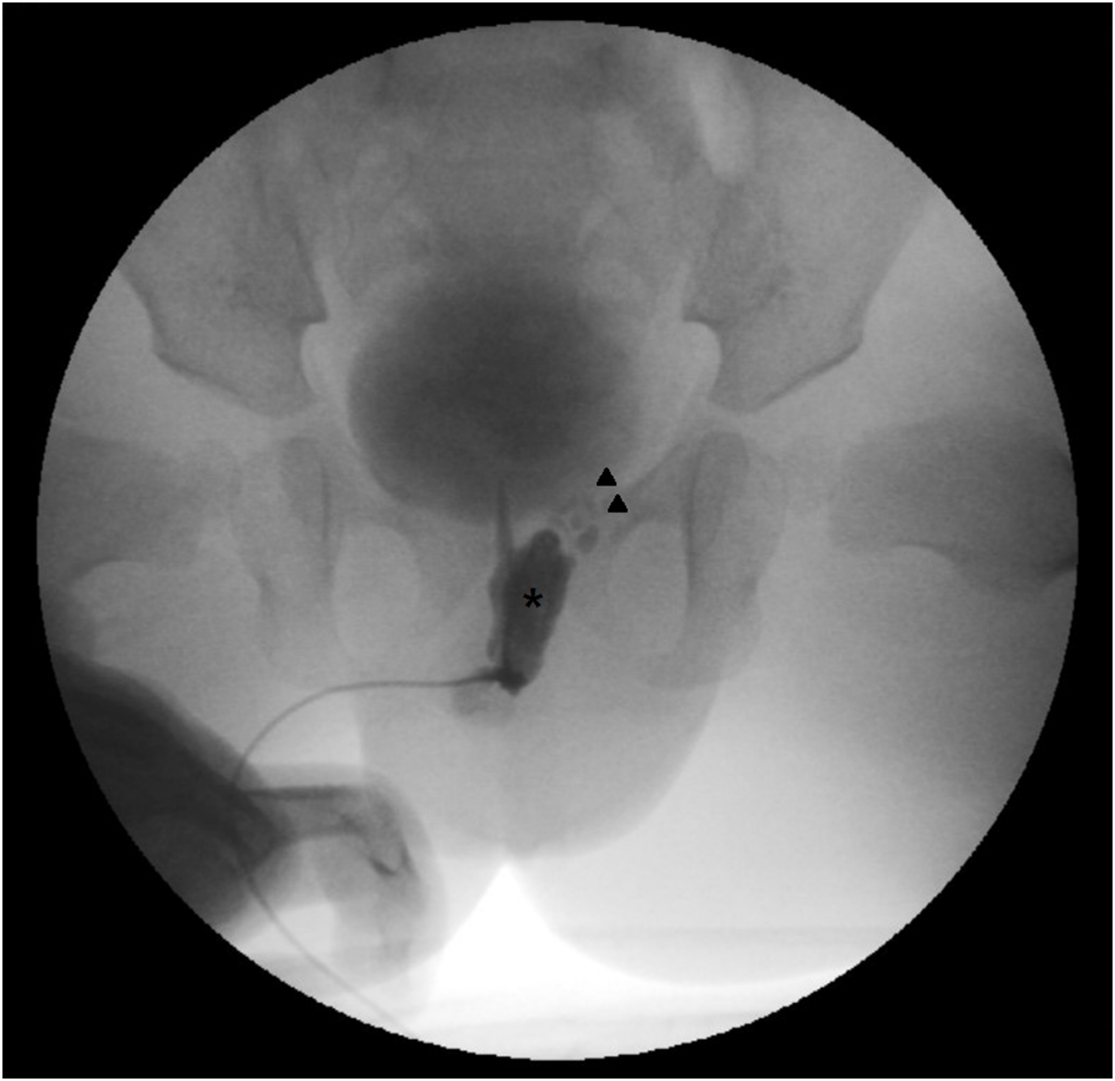
Cystic dilated prostatic utricle found in 11 month old boy after 3^rd^ episodes of left acute epididymitis. Left ejaculatory duct was inserted not into the urethra, but into the prostatic utricle. Left vasectomy was performed in this patient. *Arrow head: left ejaculatory duct, Asterisk: cystic dilated prostatic utricle.

Antibiotics were used to treat all patients with AE. Vasectomy was recommended for patients with recurrent AE with an accompanying GUA. Nineteen patients (10.1%) underwent a vasectomy, including 16 unilateral vasectomies and three bilateral vasectomies (Fig 3).

The initial vasectomy was performed at scrotal level in 14 patients and inguinal level in two patients. In the other three patients, vasectomies were performed concurrently with other operations: two with utricle excisions and one with an ureteroneocystostomy. Following the vasectomy, five patients developed funiculitis. Among the funiculitis patients, additional distal-level vasectomies were performed in four patients. Vasectomies near the ureterovesical junction were performed in three of these patients due to inguinal abscesses developing at the scrotal or inguinal level following vasectomy, and the remaining patient had an additional inguinal-level vasectomy following an initial scrotal-level vasectomy.

Prostatic utricle anomalies were excised in four of the 27 patients. In two of the patients, the vas was inserted into the utricle, and these patients underwent a simultaneous utricle excision and vasectomy. In the other two patients, the utricle was partially excised laparoscopically to spare the vas. However, recurrent AE developed in both patients after the partial utricle excisions, and subsequently, scrotal vasectomies were performed.

## Discussion

While there are a few reports describing pediatric AE, they primarily focus on whether evaluating for an accompanying GUA is necessary. Previously, Cappele et al, recommended further GUA evaluation after recurrence or after epididymitis with accompanying bacteriuria.[2] Recently, Redshaw et al. reported that bacteriuria is a risk factor for existing GUA.[4] Notably, both reports mixed pre- and postpubertal AE results. Because postpubertal AE is primarily caused by sexually transmitted disease, the clinical characteristics are quite different from that of prepubertal AE.[2] A few reports separated their analysis of prepubertal AE. For example, Siegel et al. evaluated 47 AE patients under 19 years of age, including 17 with prepubertal AE.[3] They reported eight GUAs among the 17 patients evaluated. Merlini et al. analyzed 25 AE patients under 15 years of age.[5] In that study, they observed eight GUAs in the 11 patients that developed AE during infantile period, and three GUAs in the 14 prepubertal patients that developed AE after that period. Because of the high incidence of accompanying GUA, the authors encouraged aggressive GUA evaluation in cases of prepubertal AE.

However, GUAs are frequently detected prior to AE development. There are several medical conditions that increase a patient’s susceptibility to AE development, such as neurogenic bladder, congenital ejaculatory duct anomalies, and urethral abnormalities including hypospadias, imperforated anus with urethrorectal fistula, and stricture.[6] With the exception of ejaculatory duct anomalies, these conditions are detected easily. In this study, 89.1% of accompanying GUAs were diagnosed prior to the first AE episode, because the patients had visible genital anomalies or prenatally detected upper urinary tract anomalies.

Therefore, we evaluated the differences in clinical characteristics and the recurrence rate between first-time AE patients with a known GUA and those with an unknown GUA status. We observed significant differences in several clinical variables. This indicates that different management strategies should be used depending on the situation. In the case of known GUAs, clinicians should focus on the existence of additional undiagnosed anomalies, the incidence and risk factors for AE recurrence, and proper treatment. Because the existence of utricle abnormality is the only observed risk factor for recurrence, clinicians should evaluate for undiagnosed CDPUs.

**Fig 4:**
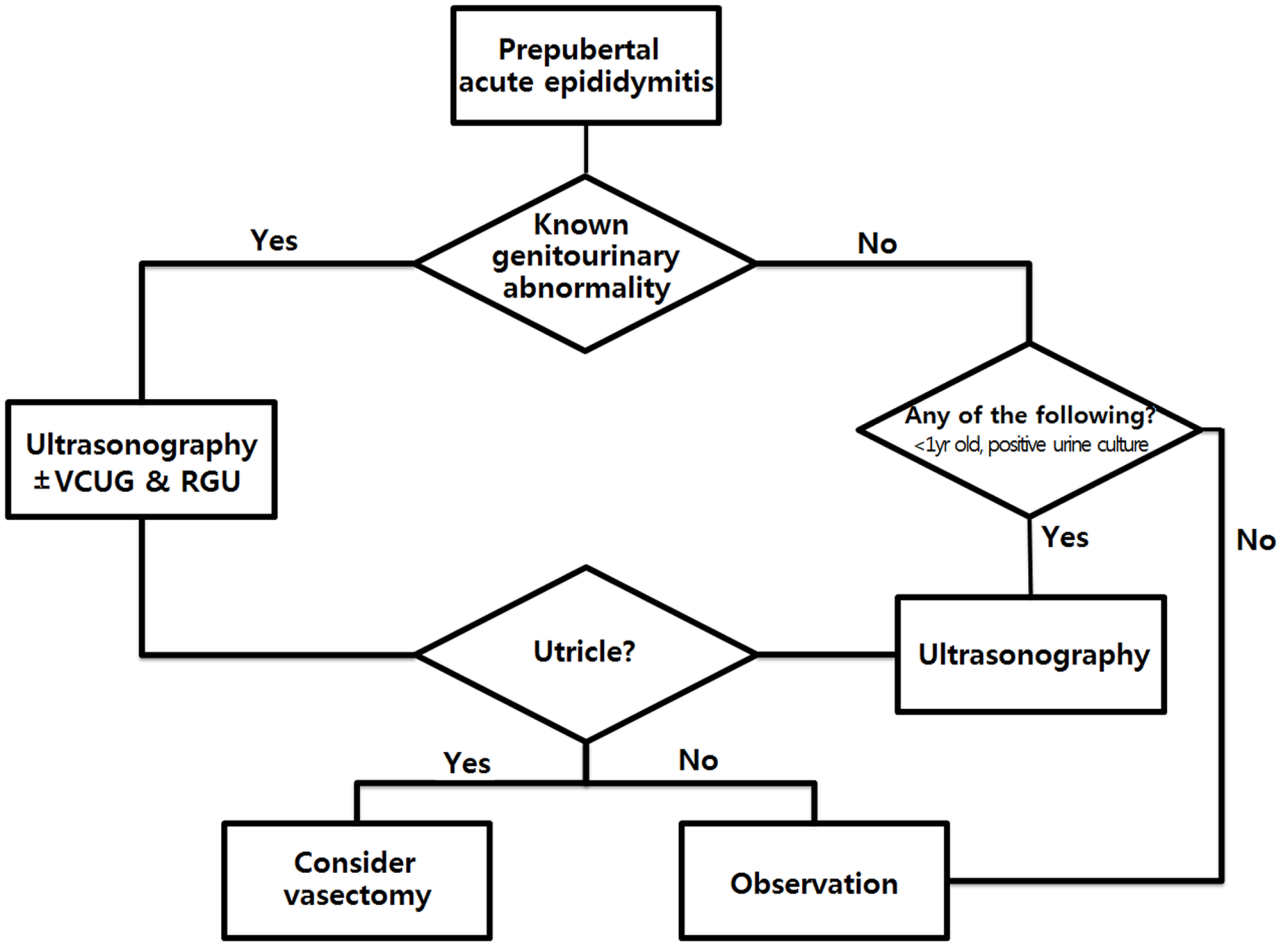
Algorithm for managing the treatment of prepubertal epididymitis; VCUG, voiding cystourethrography; RGU, retrograde urethrography.

To detect prostatic utricle abnormalities, ultrasonography, VCUG, and RGU are commonly used. Previously, Kojima et al. reported 75% and 83% utricle abnormality detection rates in hypospadias patients using ultrasonography and RGU, respectively.[7] They recommended using non-invasive ultrasonography as an initial imaging method during diagnosis. Our detection rates are consistent with those of their study, and we agree with their recommendation. Therefore, when a GUA has been diagnosed previously, we suggest using ultrasonography first and proceeding to VCUG and/or RGU in the event of a negative ultrasonography result (Fig 4).

By contrast, when the GUA status in the first-time AE patient is unknown, the probabilities of an existing, undetected anomaly or recurrence are very low. In unknown GUA status patients, we recommend evaluating for GUAs on a limited basis, e.g., when the patient is less than 1-year-old or has a positive urine culture. Cappele et al. evaluated 38 pediatric epididymitis patients with no history of prior urological issues and found GUAs in 18% of patients.[2] In their study, patients less than 2 years old and bacteriuria were not risk factors for co-existing anomalies. Their results contradict those of our study, with respect to prevalence (4.3%) and risk factors (early age and positive urine culture). These conflicting results could be related to the differences in the number of enrolled patients and their age distribution. In addition, our retrospective study did not perform imaging studies on all the patients in the unknown GUA status group, which could lead to false negatives in this patient group. However, only four patients (3.0%) of the 134 patients presumed to be negative for GUA had a second AE episode, and none experienced a third event during the study period. Therefore, false negatives are probably not clinically significant, at least with respect to the unknown GUA status group.

The question of whether to perform a vasectomy in recurrent cases is an important issue. However, this question could not be answered with respect to testicular or epididymal function, because the extent of long-term testicular or epididymal damage after a single AE episode has not been well evaluated. Moreover, a vasectomy can also cause a decrease in testicular function.[8] Therefore, we should consider the clinical course and the lessons learned through clinical experience. In the known GUA group, the recurrence rate was high, especially in patients with CDPUs. Second, third, and fourth AE episodes were observed in 90.5%, 87.1%, and 87.5% of patients, respectively, when they were not treated with a vasectomy following an episode. Thus, when patients have CDPUs in addition to other pre-diagnosed GUAs, vasectomies should be considered. In contrast, recurrence rate was very low in the unknown GUA status group. This indicates that when there are risk factors for an accompanying GUA, the utricle should be evaluated using ultrasonography. A vasectomy should only be considered after a CDPU is detected.

Selecting the proper site for the vasectomy is another important matter; however, there is no definitive course for making this decision. Kajbafzadeh et al. discussed the matter of vasectomy reversal in patients with recurrent AE, suggesting that vasectomies at scrotum level may be preferable.[9] However, these patients often have an anatomical anomaly, such as CDPUs, and the dismembered vas may not be the only source of long-term fertility problem in these cases. Moreover, in our study, funiculitis developed after the initial vasectomy requiring an additional vasectomy at a more distal level. Considering these issues, the inguinal canal could be an option as an initial vasectomy site.

We used antibiotics even in patients with negative urine culture to prevent secondary infection. According to the literature review by Cristoforo, more than 80% of patients with AE were treated antibiotics.[10] However, routine use of antibiotics in patients with AE is not recommended. Santillanes et al. recommended withholding antibiotics until positive urine culture when the initial urinalysis is clean.[11] Our study could support their recommendation especially in patients with unknown GUA status group.

There has been some controversy regarding the age distribution of AE patients. Gislason et al. reported that AE occurred rarely during the infantile period.[12] However, other papers showed a bimodal distribution of AE cases, with the highest incidence occurring in the infant and teenage periods.[2, 13] In this study, there was a large difference in age distribution between the known GUA and unknown GUA status groups. In the known GUA group, the first AE occurred most frequently in 0–1 year olds. However, in the unknown GUA status group, the first AE occurred most frequently in 9–10 year olds. Because previous studies have not distinguished between known and unknown GUA status in their evaluations of AE patients, they may have contradicting results.

There have been some reports of the presence of utricle in hypospadias patients. Ikoma et al. reported that enlargement of the utricle was present in 31.5% of hypospadias patients and Devine et al. reported that the incidence is related to the type of hypospadias.[14, 15] As AE developed after hypospadias repair in most of cases in our study, ‘repair’ of hypospadias might be a risk factor for the development of AE. However, our study did not perform a risk factor analysis on the development of AEs. Instead, we performed a risk factor analysis on the recurrence of AE in the known GUA group. As a result, we found that the presence of utricle was a risk factor rather that of hypospadias. Whether the ‘repair’ of hypospadias is a risk factor could be analyzed by further cohort study.

This study has a few limitations. The retrospective design introduces the potential for selection bias. Another major limitation is that patients with testicular appendix torsion might be included in our study especially in the unknown GUA status group. Testicular appendix torsion is a major differential diagnosis of AE in this age group. Although we excluded patients with clear signs of testicular appendix torsion, all testicular appendix torsion could not be diagnosed with physical examination and ultrasonography. And therefore, severe type of appendix torsion showing localized enlargement and epididymal tenderness during the physical and increased epididymal size and blood flow on Doppler ultrasonography might be included in this study especially in unknown GUA status group. We could not distinguish them clearly at this moment due to the retrospective nature of this study. In addition, urine culture and blood test like C-reactive protein were not performed in all patients, and the prostatic utricle detection methods were not controlled. Vasectomy as a treatment method was not controlled, because the parents decided whether to proceed.

To date, the largest study analyzing AE was that of Redshaw et al. They analyzed 252 AE patients, including 68 patients less than 10 years of age. They highlighted the necessity for risk factor analysis with respect to recurrent AE as a future study goal. In our study, we included 189 prepubertal patients and analyzed the risk factors for recurrence. To our knowledge, this is the largest study analyzing prepubertal AE and the first to investigate recurrence risk factors. In addition, while previous studies focused primarily on the presence of a GUA, we analyzed the different characteristics and clinical courses based on whether there was a pre-diagnosed GUA. Further controlled studies with long-term follow up, especially with respect to testicular and epididymal function including fertility, would provide valuable information for future treatment decisions.

## Conclusions

Prepubertal AE frequently accompanies GUA, and most GUAs are diagnosed prior to AE development. Clinicians should consider different treatment approaches based on whether the AE patient has been diagnosed with a GUA previously, because the clinical characteristics and the recurrence rate are significantly different.

## Supporting information

S1 File We attached our data sets. (XLS)
